# Assessment of the nutrition environment in rural counties in the Deep South

**DOI:** 10.1017/jns.2018.18

**Published:** 2018-10-25

**Authors:** James M. Shikany, Tiffany L. Carson, Claudia M. Hardy, Yufeng Li, Samara Sterling, Sharonda Hardy, Cordie M. Walker, Monica L. Baskin

**Affiliations:** 1 Division of Preventive Medicine, School of Medicine, University of Alabama at Birmingham, 1720 2nd Avenue South, Birmingham, AL 35294-4410, USA; 2 Comprehensive Cancer Center, University of Alabama at Birmingham, 1720 2nd Avenue South, Birmingham, AL 35294-3300, USA; 3 Department of Nutrition Sciences, School of Health Professions, University of Alabama at Birmingham, 1675 University Boulevard, Birmingham, AL 35294-3360, USA

**Keywords:** African Americans, Food quality, Nutrition surveys, Rural populations, CARES, Cancer Survivors And Relatives Eating/Exercise Support, NEMS-S, Nutrition Environment Measures Survey in Stores

## Abstract

The nutrition environment, including food store type, may influence dietary choices, which in turn can affect risk of obesity and related chronic diseases such as CHD, diabetes and cancer. The objective of the present study was to elucidate the extent to which healthy foods are available and affordable in various rural food outlets. A subset of the nutrition environment was assessed using the Nutrition Environment Measures Survey in Stores (NEMS-S). The NEMS-S instrument assessed the availability and price of healthy foods (e.g. low-fat/non-fat milk, lean meats and reduced-fat dinner entrées) compared with less healthy counterparts (e.g. whole milk, non-lean meats and regular dinner entrées). The NEMS-S also assessed the quality of fresh fruits and vegetables. Availability, prices and quality of healthy foods were compared between grocery stores (*n* 24) and convenience stores (*n* 67) in nine rural counties in Alabama. Mean availability subscale score (possible range 0 to 30; higher score indicates a greater number of healthier foods were available) for grocery stores was 22·6 (sd 8·1), compared with 6·6 (sd 5·2) in convenience stores (*P* < 0·0001); and mean price subscale score (possible range −9 to 18; higher score indicates that healthier options were less expensive than the less healthy options) for grocery stores was 2·4 (sd 2·7), compared with 0·7 (sd 1·2) in convenience stores (*P* = 0·0080). Mean total NEMS-S score (possible range −9 to 54) in grocery stores was 29·8 (sd 10·9) compared with 7·3 (sd 7·1) in convenience stores (*P* < 0·0001). Both grocery and convenience stores could be strategic points of intervention to improve the nutrition environment in the counties that were surveyed.

While generally it is agreed that the problem of obesity has risen to epidemic proportions, the specific factors that are implicated in the development of obesity are still under investigation^(^^1^^)^. Obesity, which is mediated in part by dietary behaviours, has been linked to the development of heart disease, diabetes, certain cancers and a host of other diseases^(^^2^^)^. Although proper education about healthy foods can influence an individual's choices, dietary behaviour also may be influenced by the availability of healthy food choices in food outlets like grocery and convenience stores^(^^3^^)^. In attempting to pinpoint specific contributors to obesity and related chronic diseases (CHD, diabetes and cancer), individual responsibility cannot be ignored; yet the nutrition environment to which one is exposed may, to a large degree, influence the dietary choices that a person makes, which in turn can affect the risk of obesity and related chronic diseases^(^^3^^)^.

The prevalence of obesity is higher in lower-income and African American communities than in the general population^(^^4^^,^^5^^)^. In North America, the general finding is that in these communities, the availability of healthy food choices (e.g. fruit, vegetables, wholewheat breads) is less than in other areas^(^^5^^)^. Convenience stores, which are less likely to offer healthy options like low-fat or low-Na foods^(^^6^^–^^8^^)^, are also more abundant than grocery stores in these locations^(^^5^^)^. Changes in dietary behaviours have been observed when the nutrition environment changes. African American residents increased fruit and vegetable intake by 32 % when there was availability of healthy food options in the local supermarket^(^^9^^)^. Furthermore, availability of supermarkets in neighbourhoods was associated with lower obesity risk^(^^9^^)^.

Disparities in obesity may also be seen by geographical area; specifically, a higher prevalence of obesity has been noted in rural than in urban populations^(^^10^^)^. Little is known about the nutrition environments in rural communities that may predispose residents to higher obesity risk than their urban counterparts. The objective of the present study was to elucidate the extent to which healthy foods are available and affordable in various food outlets in rural counties in Alabama.

## Methods

### Geographical areas assessed

A subset of the nutrition environment was assessed in nine rural counties in Alabama. The counties were part of an ongoing academic–community partnership to reduce or eliminate cancer disparities between African Americans and Caucasians living in the Deep South region of the USA^(^^11^^–^^13^^)^. Selected counties were from the Alabama Black Belt, a region characterised by its dark, rich soil and associated agricultural heritage: Barbour, Butler, Dallas, Greene, Hale, Lowndes, Macon, Marengo and Pickens.

### Nutrition environment assessment

The nutrition environment of the targeted counties was assessed using the Nutrition Environment Measures Survey in Stores (NEMS-S; https://www.med.upenn.edu/nems/docs/NEMS_S_Detailed.pdf)^(^^14^^)^. The NEMS-S instrument assesses the availability, price and quality of healthy foods that are available in retail food stores relative to less healthy choices. The original instrument has high inter-rater reliability (0·84–1·00) and test–retest reliability (0·73–1·00)^(^^14^^)^. Some of the healthy items assessed include fruit and vegetables (fresh, canned and frozen), low-fat/non-fat milk, lean meats and reduced-fat dinner entrées. The availability and prices of these food items were recorded and compared with the prices of their less healthy counterparts, including whole milk, non-lean meats and regular dinner entrées. Grocery stores and convenience stores were compared to see the extent to which the availability, prices and quality of healthy foods varied by food store type.

The NEMS-S instrument includes three subscale scores (availability, price and quality) and a total score (sum of subscale scores). Availability scores can range from 0 to 30, with higher scores representing a greater number of healthier foods available. Price scores can range from −9 to 18 and are derived by comparing the price of the healthier option with the less healthy/regular option. Higher points were assigned when the healthier option was less expensive than the less healthy/regular option. Finally, fresh fruit and vegetables were assigned a quality score by subjective rating of whether greater than 50 % of the item was acceptable (*v.* unacceptable, characterised as rotten, bruised, discoloured, or otherwise unappealing). This subscale score can range from 0 to 6. Accordingly, the total NEMS-S score can range from −9 to 54.

The NEMS-S instrument was administered by the study programme coordinator and Cancer Survivors And Relatives Eating/Exercise Support (CARES) coordinators. CARES coordinators were local lay individuals who were trained by research staff to lead outreach and research activities as a part of the Deep South Network. These individuals were paid, part-time staff of the University of Alabama at Birmingham who worked remotely from their local communities throughout the state. CARES coordinators were utilised to conduct the NEMS-S assessment because of their familiarity with their communities and established relationships in working with academic institutions to conduct community-based research.

Training of CARES coordinators for the NEMS-S audits took place in a county that was central to each of their nine counties. The training was led by the study programme coordinator who had previously been trained by a certified NEMS-S trainer in a day-long session that included a 3-h PowerPoint presentation with samples of each questionnaire, followed by a field trip to a local grocery store in the metropolitan Birmingham, Alabama area for real-life practice in administering the instrument. The field practice was conducted in the county where the training occurred since it was not a county that would be assessed as a part of the study. In preparation for the local training, research staff provided a sample packet for the CARES coordinator that included: grocery/convenience store assignments, two sample surveys (one to keep and one to use during the field training) and a sample letter for the store manager.

Through a combination method of geographic information systems (GIS) mapping and county health department databases, investigators and research staff compiled a list of grocery and convenience stores in each targeted county. This list was sent to the CARES coordinator to conduct ‘ground truthing’ to confirm the exact location of the store in the county and to determine if the store was still in business. During ground truthing, CARES coordinators also reported if there were any new stores present that did not appear in either the GIS or health department databases. This process provided final verification of all stores in the local area. Once ground truthing was completed, research staff compiled the final list of stores in the area. Members of the investigative team and research staff met to systematically determine the subsample of stores to be surveyed based on the population density of local residents.

The study programme coordinator from the central office travelled to each county and partnered with the CARES coordinator to complete the assessments for each of the stores listed on the final store list. A NEMS-S survey packet was created for each store to be assessed. This packet included the store list and identifying codes assigned to each store, the NEMS-S forms and sufficient copies of the letters to the store managers.

During the survey, the programme coordinator and CARES coordinator recorded the availability of healthy food items relative to less healthy choices in the grocery and convenience stores that they visited. They recorded the prices of the items, along with the quality (acceptable or unacceptable) of the produce examined. Data collection was completed within 2–3 weeks of assignment.

The study programme coordinator conducted quality assurance reviews as assessments were completed. This included providing help in troubleshooting any problems that were encountered with store visits. Surveys also were reviewed to ensure that all items were completed; writing was legible; all items were answered with a ‘yes’, ‘no’ or ‘N/A’ (not applicable); and all ‘Measure Complete’ fields were filled in correctly. Once the completeness and accuracy of the documents were confirmed, forms were submitted to the data manager in the research office for data entry.

### Statistical analysis

The availability, price and quality of healthy foods in convenience and grocery stores were directly assessed using the NEMS-S instrument, which assessed ten categories of foods: milk, fruit, vegetables, meats, frozen dinners, baked goods, beverages, bread, chips (crisps) and cereals. The tool Scoring Systems for NEMS Store Measures was used to assist in calculating subscale scores and total NEMS-S scores^(^^14^^)^. For each item (milk, fruit, etc.), there are different positive and negative point values to assign for various factors in availability, price and quality (fruit and vegetables only). After completing the scoring algorithm, we calculated the subscale scores for availability, price and quality, and then added these to obtain the total score.

We report the availability, price, quality and total score for grocery and convenience stores in the nine targeted counties. Descriptive analysis was conducted to describe the characteristics of the counties. We used two-group Student's *t* tests to compare the continuous availability and price measures of more healthful foods between the two store types (convenience and grocery), respectively. Fisher's exact tests or χ^2^ tests were used to compare the categorical availability measures by store type. A *P* value less than 0·05 was considered statistically significant. Analyses were conducted using SAS statistical software, version 9.3 (SAS Institute).

## Results

Assessments were conducted between May 2013 and November 2015.

### County characteristics

A total of twenty-four grocery stores and sixty-seven convenience stores were surveyed in the nine rural Alabama counties. The mean population in the nine rural Alabama counties included in the NEMS-S assessment was 20 346 (sd 9197), with a range of 8553 to 41 711 (Table 1). The mean population density in the nine counties was 26·2 (sd 9·1) persons per square mile, with a range of 14·0 to 35·2 person per square mile. African American residents comprised a mean of 60·6 (sd 15·0) % of the population in the included counties, with a range of 40·2 to 81·5 %. The median annual household income was $29 918 (interquartile range 26 519, 30 724) in the counties, with a range of $24 226 to $35 079. Finally, the mean proportion of the population below the federal poverty level^(^^15^^)^ in the counties was 28·4 (sd 3·4) %, with a range of 24·0 to 35·5 %.

**Table 1.** Characteristics of the rural Alabama counties included in the nutrition environment assessment, 2010–2014*

County	Population	Population density (persons per square mile)	African American (%)	Median household income ($)	Population below poverty level (%)
Barbour	26 887	31·0	47·6	32 911	26·7
Butler	20 296	27·0	44·0	29 918	28·4
Dallas	41 711	44·8	69·5	26 519	35·5
Greene	8553	14·0	80·5	24 226	32·9
Hale	15 184	24·5	58·1	30 051	26·6
Lowndes	10 580	15·8	72·9	26 230	26·7
Macon	19 425	35·2	81·5	30 724	27·3
Marengo	20 110	21·5	51·0	35 079	24·0
Pickens	20 365	22·4	40·2	28 741	27·2
Overall					
Mean	20 346	26·2	60·6	29 918†	28·4
sd	9197	9·1	15·0	26 519, 30 724‡	3·4

* Data from the United States Census Bureau.

† Median.

‡ Interquartile range.

### Availability subscale score

Availability of healthy options in each of the categories of foods surveyed was compared between convenience stores and grocery stores in the nine counties (Table 2). Generally, healthy choices were available in a significantly higher proportion of grocery stores compared with convenience stores (*P* < 0·0001 for all), with the exception of diet soda and 100 % fruit juice, which were available in almost all grocery and convenience stores. Fruit was available in all grocery stores compared with just over half of convenience stores, and most convenience stores in which fruit was available included no more than four types of fruit, while at least ten varieties of fruit were available in more than half of grocery stores. A similar pattern was observed for the availability of vegetables. Several healthy foods were available in virtually all of the grocery stores, but also in a meaningful proportion (22–40 %) of convenience stores (e.g. low-fat or non-fat milk, 100 % whole-grain bread and healthier cereal). Other healthy foods were available in most or all of the grocery stores, but in none or almost none of the convenience stores (e.g. low-fat or fat-free hot dogs, reduced-fat frozen dinners and low-fat baked goods). Finally, a small number of healthy foods (lean ground beef and baked or low-fat chips (crisps)) were available in a third to a half of the grocery stores, but in none or very few of the convenience stores. Considering all categories of foods surveyed, the mean availability subscale score (possible range 0 to 30) for grocery stores was 22·6 (sd 8·1), compared with 6·6 (sd 5·2) in convenience stores (*P* < 0·0001).

**Table 2.** Availability of healthy foods comparing convenience with grocery stores in rural Alabama counties (May 2013–November 2015) (Numbers and percentages)

	Convenience stores (*n* 67)		Grocery stores (*n* 24)		
Food	Number	%	Number	%	P*
Milk, low-fat† or non-fat	27	40·3	24	100·0	<0·001
Fruit, any	39	58·2	24	100·0	<0·001
Fruit, number of varieties					
0	28	41·8	0	0·0	<0·001
1–4	37	55·2	0	0·0	
5–9	2	3·0	11	45·8	
10–11	0	0·0	13	54·2	
Vegetables, any	52	77·6	24	100·0	<0·001
Vegetables, number of varieties					
0	15	22·4	0	0·0	<0·001
1–4	36	53·7	0	0·0	
5–9	16	23·9	0	0·0	
10–13	0	0·0	24	100·0	
Ground beef, lean	0	0·0	8	33·3	<0·001
Hot dogs, low-fat or fat-free	2	3·0	19	79·2	<0·001
Frozen dinners, reduced-fat	0	0·0	22	91·7	<0·001
Baked goods, low-fat	2	3·0	18	75·0	<0·001
Soda, diet	65	97·0	24	100·0	0·69
Fruit juice, 100 %	65	97·0	24	100·0	0·69
Bread, 100 % whole-grain	15	22·4	24	100·0	<0·001
Whole-grain bread: ≥2 varieties	2	3·0	23	95·8	<0·001
Chips (crisps), baked or low-fat	3	4·5	13	54·2	<0·001
Cereal, healthier	26	38·8	24	100·0	<0·001

* *P* values from Fisher's exact test or χ^2^ statistic.

† 1 or 2 % fat.

### Price subscale score

For foods available in a substantial proportion of both grocery and convenience stores, we compared whether the price for the healthy version of each food was lower than, the same as, or more expensive than the price of the regular version of the food by store type (Table 3). While low-fat or non-fat milk was the same price as whole milk in all of the convenience stores with price data, low-fat or non-fat milk was lower in cost than whole milk in more than half (58 %) of the grocery stores (*P* < 0·0001). It was found that 100 % fruit juice was the same price as sugar-sweetened juice in more than 80 % of convenience stores, but was more expensive than sugar-sweetened juice in more than 90 % of grocery stores (*P* < 0·0001). Prices for whole-grain breads tended to be the same as regular breads in convenience stores, while prices for whole-grain bread tended to be higher than regular breads in grocery stores (*P* < 0·0001). Prices for healthier cereals and regular cereals were the same in more than half of the convenience stores stocking these cereals. However, in half of grocery stores, prices for healthier cereals were lower than those for regular cereals (*P* < 0·0001). Diet soda was the same price as regular soda in almost all convenience and grocery stores (*P* = 0·674). Considering all categories of foods surveyed, the mean price subscale score (possible range −9 to 18) for grocery stores was 2·4 (sd 2·7), compared with 0·7 (sd 1·2) in convenience stores (*P* = 0·0080).

**Table 3.** Price of selected healthy foods relative to the regular version comparing convenience and grocery stores in rural Alabama counties (May 2013–November 2015) (Numbers and percentages)

Food	Convenience stores		Grocery stores		P*
	Number	%	Number	%	
Milk					
No. of stores	27		24		
Lower price for low-fat† or non-fat	0	0·0	14	58·3	<0·001
Same price for low-fat or non-fat	22	81·5	8	33·3	
Higher price for low-fat or non-fat	0	0·0	1	4·2	
Price not available	5	18·5	1	4·2	
Soda					
No. of stores	65		24		
Lower price for diet	3	4·6	0	0·0	0·67
Same price for diet	61	93·8	23	95·8	
Higher price for diet	1	1·5	0	0·0	
Price not available	0	0·0	1	4·2	
Fruit juice					
No. of stores	65		24		
Lower price for 100 %	2	3·1	2	8·3	<0·001
Same price for 100 %	54	83·1	0	0·0	
Higher price for 100 %	7	10·8	22	91·7	
Price not available	2	3·1	0	0·0	
Bread					
No. of stores	15		24		
Lower price for whole-grain	2	13·3	1	4·2	<0·001
Same price for whole-grain	9	60·0	8	33·3	
Higher price for whole-grain	3	20·0	15	62·5	
Price not available	1	6·7	0	0·0	
Cereal					
No. of stores	26		24		
Lower price for healthier	3	11·5	12	50·0	<0·001
Same price for healthier	15	57·7	3	12·5	
Higher price for healthier	0	0·0	8	33·3	
Price not available	8	30·8	1	4·2	

* *P* values from χ^2^ statistic.

† 1 or 2 % fat.

### Quality subscale score

While quality data (acceptable quality *v.* not acceptable quality) were available on all fresh fruit and vegetables in the twenty-four grocery stores, these scores were not available for most fresh fruit and vegetables in the convenience stores due to their low availability in these stores (data not shown); therefore, comparisons by store type were not possible for the most part. With this limitation taken into account, the mean quality subscale score (possible range 0 to 6) for grocery stores was 4·8 (sd 1·2), compared with 0·03 (sd 0·2) in convenience stores (*P* < 0·0001).

### Total Nutrition Environment Measures Survey in Stores score

Considering availability, price and quality subscale scores, the mean total NEMS-S score (possible range −9 to 54) in the twenty-four grocery stores was 29·8 (sd 10·9) compared with 7·3 (sd 7·1) in the sixty-seven convenience stores (*P* < 0·0001).

## Discussion

This study describes the availability, price and quality of selected food items in grocery stores and convenience stores in nine rural counties in Alabama with a high proportion of African American residents. The mean total NEMS-S score for grocery stores was significantly higher than that for convenience stores, driven by significantly higher subscale scores for all three components of the total NEMS-S score: availability, price and quality. However, total NEMS-S scores for both outlets were well below the maximum total NEMS-S score of 54, suggesting that interventions or policy actions are warranted to improve the nutrition environments as a whole in these rural communities.

Our finding regarding the greater availability of healthier food items in grocery stores compared with convenience stores is consistent with previous studies^(^^16^^–^^18^^)^. One study of a rural Florida community reported NEMS-S availability subscale scores of 30 and 3·25 for supermarkets and convenience stores, respectively^(^^16^^)^. Though we cannot directly compare the scores from the present study with the Florida study because the NEMS-S commonly allows for adaptations to ensure relevance for the targeted area, the trend for lower availability scores in convenience stores is consistent across studies. The nutrition environment of convenience stores is of particular importance because convenience stores comprise the majority of food store outlets in most rural communities, lower-income areas and communities with a greater proportion of racial/ethnic minorities^(^^6^^)^. From our ground truthing for this study, we determined that convenience stores were approximately 70 % of the stores present across the nine counties. In other research, the proportion of convenience stores is comparable, representing 70–75 % of food store outlets in rural communities^(^^16^^,^^19^^)^. Our study shows that convenience stores do provide some limited availability for healthier items including fruit and vegetables, which were available in a majority of stores.

Price subscale scores indicated that for both grocery stores and convenience stores, some healthier items had higher prices while other healthier items actually had prices that were lower than or similar to regular food options. This is consistent with other studies that reported mixed findings in terms of the pricing of healthier food items^(^^17^^)^. One study, which included both rural and urban supermarkets and corner stores, even reported that corner stores were more competitively priced than supermarkets for healthier food items^(^^18^^)^. These data are of importance because they challenge a commonly accepted belief that healthier foods are more expensive than regular food options. Convenience stores are often seen as a culprit in the obesity epidemic because they often provide easy access to high-energy, unhealthy foods at cheap prices^(^^7^^,^^8^^)^. Our findings highlight an opportunity to consider convenience stores as an efficient point of intervention. Convenience stores offered some healthier options, though less than grocery stores, and in some cases the healthier options were less expensive than or priced similarly to the regular options. Increased availability of healthier food items of acceptable quality in convenience stores may create a healthier food environment for residents of rural counties who encounter convenience stores at a much higher frequency than grocery stores.

This study has limitations, including limited external validity and lack of consideration for other sources of healthy foods. The food environment described may not be generalisable to other rural areas or more urban locations. Although there is a growing body of literature describing the food environment of rural communities, it is important to note the heterogeneity of these communities. Some of our findings are consistent with previously published literature on food environments of rural communities. However, unique characteristics of each community limit our ability to make inferences about other rural communities from these data. Additionally, this study did not include a measure of other potential food outlets such as farmers’ markets and community gardens, which increasingly are becoming a source of local fresh produce in rural areas.

### Conclusions

Increasing attention is being given to the need for multi-level (e.g. individual, community/environment) interventions to promote healthier eating in an effort to reduce obesity-related morbidity and mortality, including those related to CHD, diabetes and cancer. In order to effectively and efficiently develop and implement such interventions, it is important to characterise the current environment to identify points for future intervention. The present study suggests that both grocery stores and convenience stores could be strategic points of intervention to improve the nutrition environment in the counties that were surveyed. Future research should examine how the nutrition environment is linked to dietary behaviours of rural residents and whether changes in the nutrition environment can promote healthier dietary behaviours.
